# Identification and analysis of differentially expressed microRNAs in endometrium to explore the regulation of sheep fecundity

**DOI:** 10.1186/s12864-023-09681-y

**Published:** 2023-10-09

**Authors:** Jihong Dong, Xuecheng Jiang, Nan Liu, Hegang Li, Jinshan Zhao, Jianning He, Xiaoxiao Gao

**Affiliations:** 1 College of Animal Science and Technology, Qingdao Agricultural University, 700 Changcheng Road, Qingdao, 266109 Shandong China; 2 College of Veterinary Medicine, Qingdao Agricultural University, Qingdao, 266109 Shandong China

**Keywords:** microRNAs, Endometrium, Fecundity, Hu sheep

## Abstract

**Background:**

MicroRNAs (miRNAs) play an important regulatory role in mammalian reproduction. Currently, most studies are primarily concentrated on ovarian miRNAs, ignoring the influence of endometrial miRNAs on the fecundity of female sheep. To uncover potential regulators of sheep fecundity, RNA-seq was used to comparatively analyze miRNA expression profiles of endometrium between high prolificacy sheep (HP, litter size = 3) and low prolificacy sheep (LP, litter size = 1) with *FecB* genotype.

**Results:**

Firstly, genomic features of miRNAs from endometrium were analyzed. Furthermore, 58 differentially expressed (DE) miRNAs were found in the endometrium of Hu sheep with different litter size. A co-expression network of DE miRNAs and target genes has been constructed, and hub genes related litter size are included, such as DE miRNA unconservative_NC_019472.2_1229533 and unconservative_NC_019481.2_1637827 target to estrogen receptor α (*ESR1*) and unconservative_NC_019481.2_1637827 targets to transcription factor 7 (*TCF7*). Moreover, functional annotation analysis showed that the target genes (*NRCAM* and *NEGR1*) of the DE miRNAs were significantly enriched in cell adhesion molecules (CAMs) signaling pathway, which was related to uterine receptivity.

**Conclusion:**

Taken together, this study provides a new valuable resource for understanding the molecular mechanisms underlying Hu sheep prolificacy.

**Supplementary Information:**

The online version contains supplementary material available at 10.1186/s12864-023-09681-y.

## Introduction

Hu sheep, as an excellent local breed in China, are famous for their high prolificacy and year-round estrus [1]. A variety of marker genes have been identified, and found to be involved with litter size by affecting sheep ovulation rate, including bone morphogenetic protein receptor (*BMPR1B/FecB*), bone morphogenetic protein 15 (*BMP15/FecX*), and growth differentiation factor9 (*GDF9/FecG*) [2, 3]. In the breeding process, some Hu sheep carrying the fecundity gene (*FecB*) of high ovulation rate still reveal low prolificacy [4]. Environmental variables may have a great influence on the model of a genome wide association study [5]. Therefore, it is meaningful to research the regulatory mechanism of sheep prolificacy at the level of transcription.

MicroRNAs (miRNAs) belong to endogenous small non-coding RNAs that perform important gene-regulatory functions in animals by pairing to the mRNAs of protein-coding genes to direct their posttranscriptional repression [6]. During the past few years, studies have shown that miRNAs play important roles in female sheep reproduction [7, 8]. Especially, ovarian transcriptomic analysis revealed the interactions of miRNAs and lncRNAs were related to fecundity in different sheep [9, 10]. Furthermore, genome-wide analysis of miRNAs in the ovaries of multiple and uniparous goats were performed [11, 12], revealing that miRNAs may regulate follicular development and ovulation and consequent increase in litter size.

As we all known, ovulation and uterine receptivity are the two main controlling factors for mammalian fertility. Therefore, in addition to ovulation performance, the development and receptivity of uterus are closely related to reproductive efficiency of sheep to maintain large litters [13]. To date, 15 miRNAs in mice and 29 miRNAs have been reported in humans within various compartments of the endometrium that may potentially modulate receptivity [14]. Furthermore, miRNAs also have been found to play a pivotal role for the establishment of the proper uterine environment required for implantation [15]. Differentially expressed miRNAs in porcine ovaries have been identified as important regulators of the litter size [16]. However, there are few studies on endometrial miRNAs in the ovine fecundity.

In this study, RNA sequencing (RNA-seq) was applied to analyze the expression profiling of endometrial miRNAs between different fecundity Hu sheep, and screen the candidate miRNAs involved in high prolificacy. These results could provide useful information for under-standing the molecular basis of miRNAs in the regulation of endometrial functions, as well as the mechanism of Hu sheep prolificacy.

## Methods

This study was carried out according to the Guide for the Care and Use of Laboratory Animals (permit no. DKY2021021) prepared by the Ethics Committee of Qingdao Agricultural University.

### Animals and sample preparation

The high prolificacy sheep group (HP, *n* = 3, litter size = 3) and low prolificacy sheep group (LP, *n* = 3, litter size = 1) were selected from the nucleus herds of Hu sheep at Taizhou Sheep Industry according to their littering records (three consecutive lambing records) and polymorphism analysis of *FecB* [17], two groups of sheep in this study had similar numbers of dominant follicles and *FecB* genotype. All sheep were housed under the same conditions with free access to feed and water. Synchronous estrus of sheep was conducted according to previously described [18]. The estrous cycles of the ewes were adjusted by intravaginal progestagen sponges (30 mg; Ningbo Sansheng pharmaceutical Co., LTD, Zhejiang, China) for 11 days. Estrus was monitored by presentation of a buck fitted with an apron three times one day following sponge removal. The sheep were deeply anesthetized by intravenous administration of 3% pentobarbital sodium (30 mg/kg; Solarbio, P8410, China), and sacrificed by exsanguination in a healthy physiological stages at the second estrus (natural estrous), and endometrium was collected from the mid-part of uterine horns, and immediately frozen in liquid nitrogen for RNA extraction.

### Library preparation and sequencing

According to the manufacturer’s instruction, total RNA from 6 endometrium samples was isolated by TRIzol reagent (Invitrogen, Carlsbad, CA, USA) for RNA sequencing (RNA-seq). The purity and concentration of RNA were assessed by NanoDrop 2000 spectrophotometer (Thermo Scientific, Wilmington, DE, United States). The integrity of RNA was assessed using the RNA Nano 6000 Assay Kit of the Agilent Bioanalyzer 2100 system (Agilent Technologies, Palo Alto, CA, USA). Sequencing libraries were generated using the small RNA Sample Pre Kit (NEB, Ipswich, MA, USA). TruSeq PE Cluster Kit v4-cBot-HS (Illumia) was used to cluster the index-coded samples on a cBot Cluster Generation System according to the manufacturer’s instructions, then library preparations were sequenced on an Illumina Hiseq 2500 platform. Clean data with high quality was generated from Raw data (raw reads) of fastq format by quality control step. Clean data were obtained by removing reads containing ploy-N and low quality reads from raw data, retained 18–30 nt sequences. At the same time, the Q30 of the clean data were calculated. All the downstream analyses were based on clean data (Supplementary Table 1).

In order to filter ribosomal RNA (rRNA), transfer RNA (tRNA), small nuclear RNA (snRNA), small nucleolar RNA (snoRNA) and other ncRNA and repeats, Clean Reads respectively performed on Silva database, GtRNAdb database, Rfam database and Repbase database sequence alignment by Bowtie software [19]. Ovis_aries_v4.0_genomic was used as reference genomes for sequence alignment and subsequent analysis. The remaining reads were used to detect known miRNA and novel miRNA predicted by comparing with known miRNAs from miRBase (v21) [20], and miRDeep2 software package was used for novel miRNA [21].

### Differential expression and target gene prediction analysis

According to the values of normalized transcripts per kilobase per million reads (TPM), the differentially expressed miRNAs were identified by a p-value threshold of < 0.05 and |log2(fold change)| > 1. The data of differently expressed mRNAs was taken from our previous study [22]. According to the sheep gene sequence information of known miRNAs and newly predicted miRNAs, target gene prediction was performed using miRanda [23] and RNAhybrid [24].

### KEGG pathways and GO analysis

Kyoto Encyclopedia of Genes and Genomes (KEGG) (http://www.genome.jp/kegg) pathway analysis of target genes was performed by the KOBAS (v2.0) software [25]. Gene ontology (GO) enrichment analysis was performed by the software GOSeq (Release2.12) [26]. *P*-values (t-test) < 0.05 was indicated as significant enrichment.

### Real-time quantitative PCR

We validated the RNA sequencing data using RT-qPCR. Primers were designed online using Primer 5 (Premier Biosoft, Palo Alto, CA, USA) software. RNA (1 mg) was reverse transcribed using the miRNA 1st Strand cDNA Synthesis kit (by stem-loop; Vazyme, Nanjing, China), and the SYBR green (Vazyme, Nanjing, China) method was used for RT-qPCR. The expression levels of genes were evaluated by 2^−ΔΔCT^, and *U6* gene were used as reference genes for normalization of the miRNA data. The primer sequences used for RT-qPCR were listed in Supplementary Table 2.

### Statistical data analysis

Further analysis of RNA-seq data and graphical representations were performed using the statistical R package (R, Auckland, NZL). SPSS 19.0 software (SPSS, Chicago, IL, USA) was used to analyze the RT-qPCR data, which were presented as the means ± standard deviations (SDs). Differential gene expression levels were calculated using a t-test, and Statistical significance was defined as p < 0.05.

## Results

### Genomic features of endometrial miRNAs

**Fig. 1:**
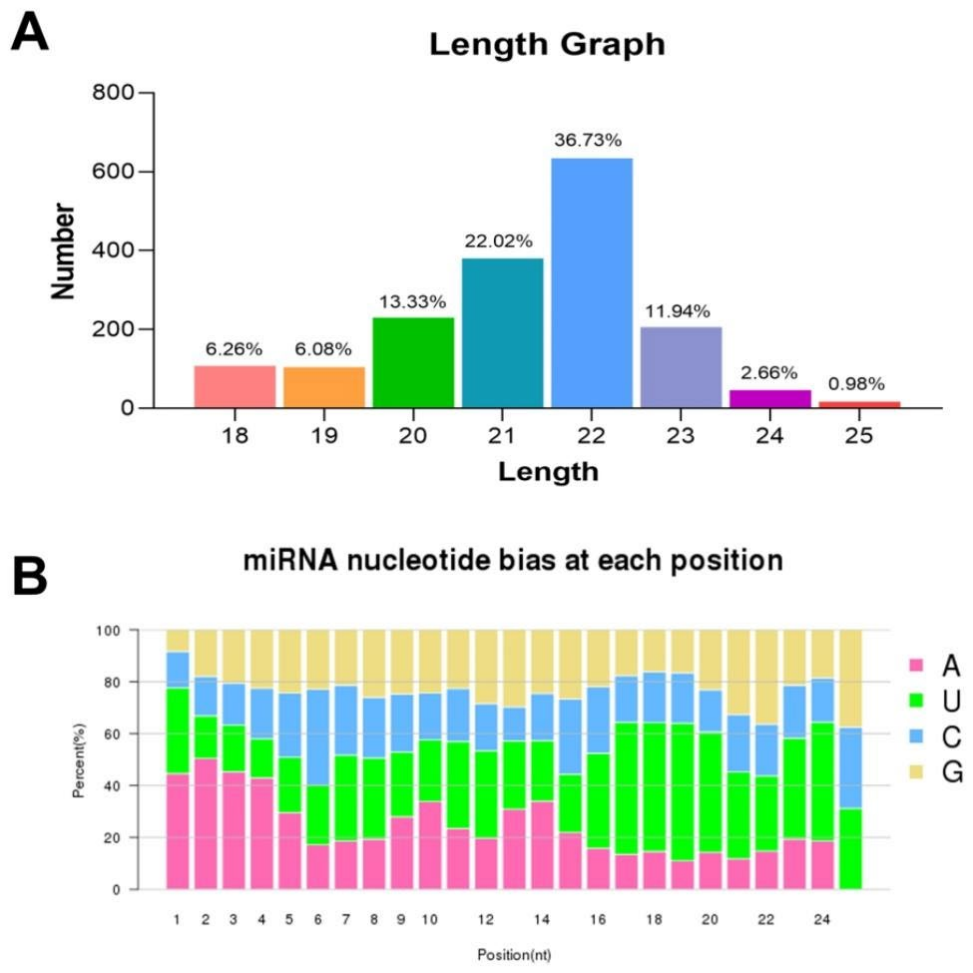
Genomic features of miRNAs from sheep endometrium. **(A)** Length distribution of miRNAs in the sheep endometrium libraries. **(B)** Nucleotide bias distribution of miRNAs.

A total of 1726 unique miRNAs were screened from sheep endometrium, result of length distribution analysis showed that most miRNAs ranged from 21 to 22nt. The percentage of the 22nt miRNAs in the total miRNAs was 36.73% (Fig. 1A). Analysis of nucleotide bias distribution showed that the proportion of base to U is the largest, especially in the 16–24 nt miRNA, followed by base A, and G and C. In the first proportion of miRNA, (A + U) was found in most miRNAs with a percentage of 77.58% in average (Fig. 1B).

### Screening of differentially expressed miRNAs

**Fig. 2:**
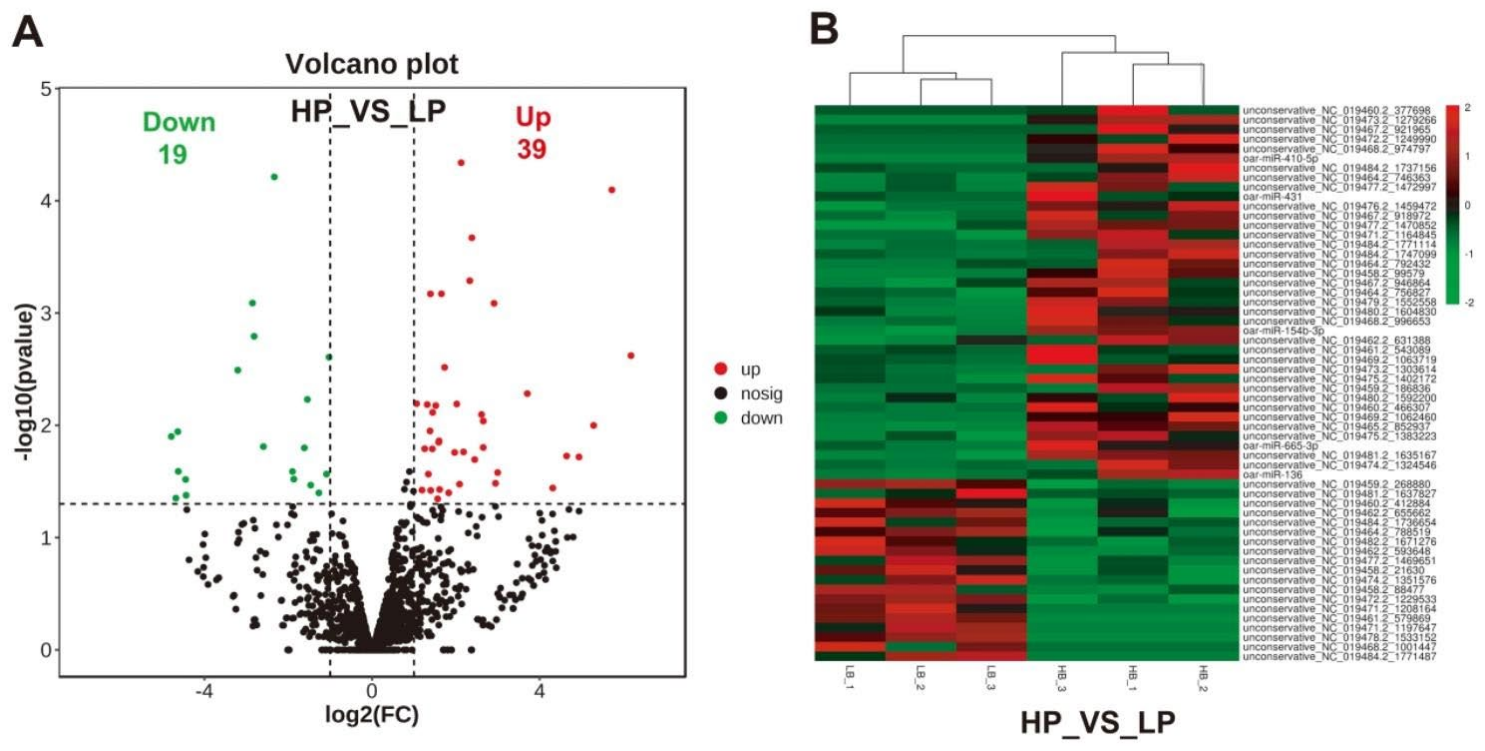
Analysis of differentially expressed miRNAs. **(A)** Differentially expressed miRNA volcano diagram. **(B)** Differentially expressed miRNA cluster. HP, high prolificacy sheep; LP, low prolificacy sheep

Further analysis identified 58 significant differential expression miRNAs (P < 0.05), including 39 up-regulated and 19 down-regulated miRNAs in high prolificacy samples compared with low prolificacy samples (Fig. 2A). In the up-regulated miRNAs, 5 known miRNAs (oar-miR-136, oar-miR-154b-3p, oar-miR-410-5p, oar-miR-431, oar-miR-665-3p) and 7 specific miRNAs expression were discovered in HP group (unconservative_NC_019478.2_1533152, unconservative_NC_019484.2_1771487, unconservative_NC_019471.2_1197647, unconservative_NC_019461.2_579869, unconservative_NC_019471.2_1208164, unconservative_NC_019468.2_1001447, unconservative_NC_019467.2_942834),

In the down-regulated miRNAs, 6 specific miRNAs expression were discovered in LP group (unconservative_NC_019473.2_1279266, unconservative_NC_019460.2_377698, unconservative_NC_019467.2_921965, unconservative_NC_019468.2_974797, unconservative_NC_019472.2_1249990, oar-miR-410-5p) (Fig. 2B; Supplementary Table 3).

**Fig. 3:**
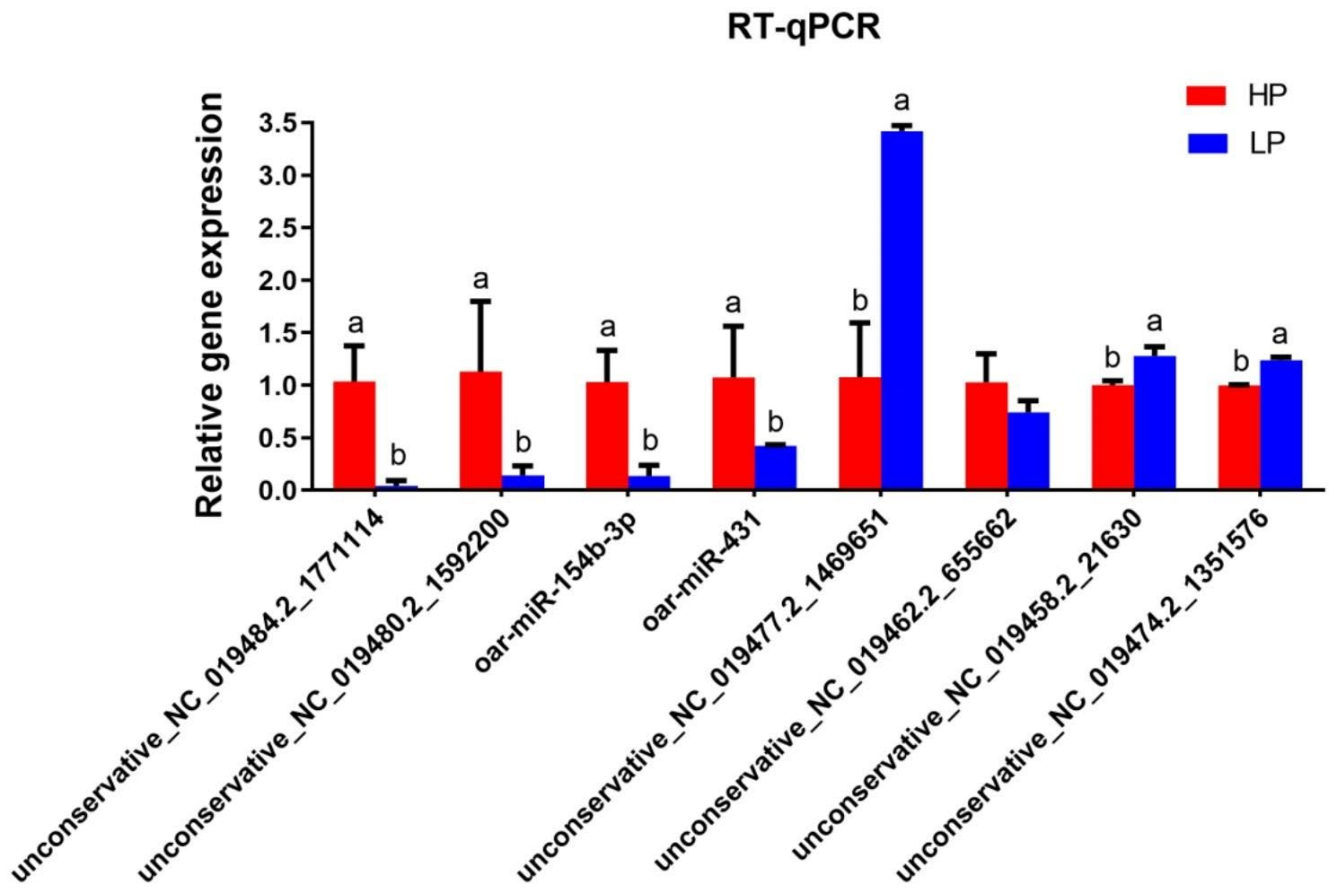
Validation of RNA-seq results by using RT-qPCR. The values are presented as means ± SDs. Means with different letters indicate significant differences (P < 0.05)

In order to verify the accuracy of the differentially expressed miRNA by RNA-seq, eight differentially expressed miRNAs were randomly selected for RT-qPCR. We found that the overall trend of this data was the same as our RNA-seq data (Fig. 3).

### Interaction network analysis of differentially expressed miRNAs and mRNAs

**Fig. 4:**
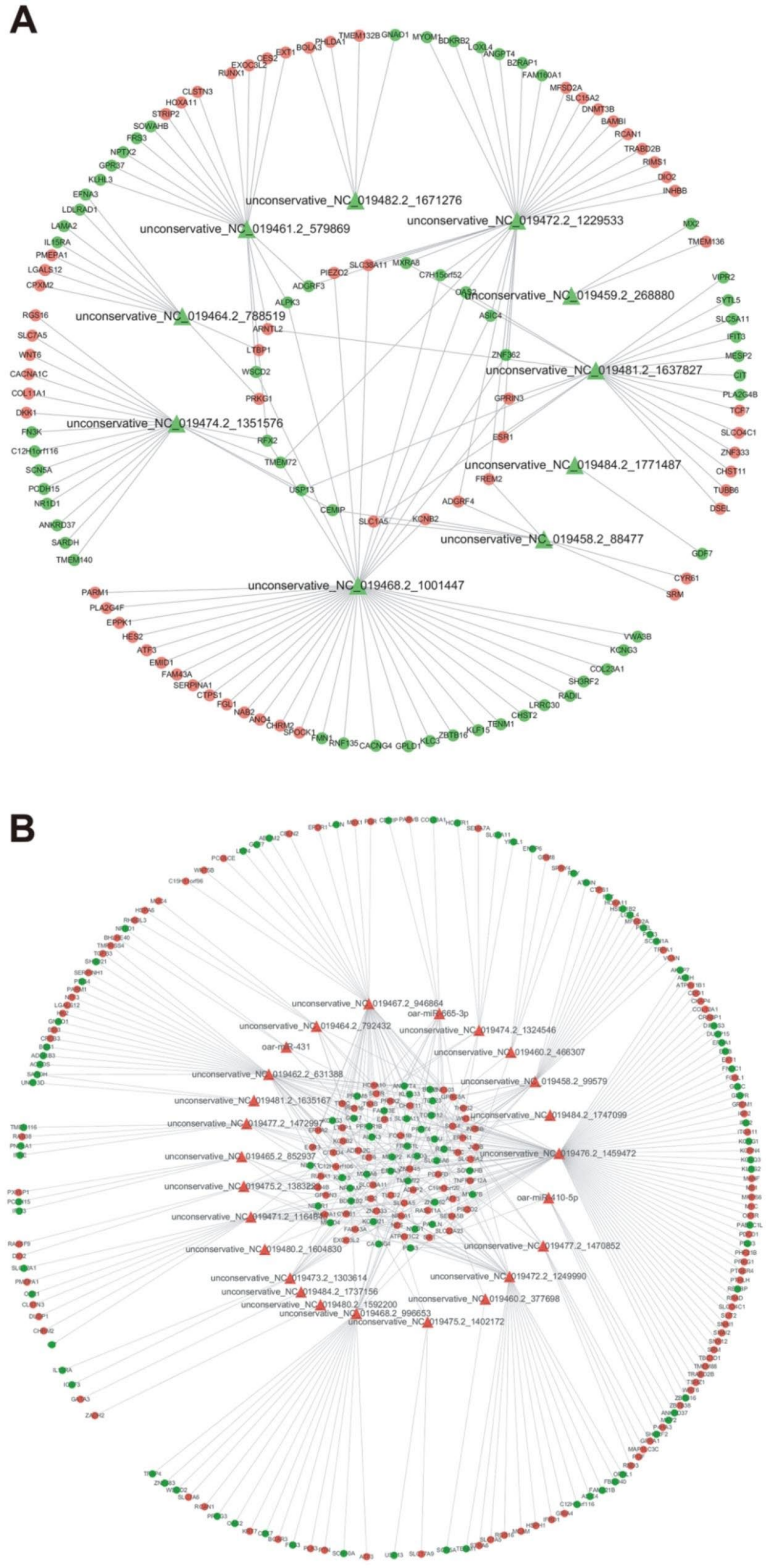
The interaction network of miRNAs-mRNAs in the comparison. **(A)** The target gene prediction of down-regulated miRNAs in the comparison. **(B)** The target gene prediction of up-regulated miRNAs in the comparison. red triangle: up-regulated miRNA; green triangle: down-regulated miRNA; red circle: up-regulated mRNA; green circle: down-regulated mRNA.

The targets of 10 down-regulated miRNAs (Fig. 4A) and 25 up-regulated miRNAs (Fig. 4B) were identified from high prolificacy sheep. A total of 538 miRNA-mRNA pairs were predicted in the HP vs. LP comparison (Supplementary Table 4). Among these miRNA-mRNA pairs, 258 pairs were negatively correlated in expression. In the network, the core gene estrogen receptor 1 (*ESR1*) was targeted by 2 miRNAs (unconservative_NC_019472.2_1229533 and unconservative_NC_019481.2_1637827). Furthermore, miRNA unconservative_NC_019481.2_1637827 also targeted to *TCF7*, which might play crucial roles in the molecular mechanism of the sheep prolific trait.

### Pathway enrichment analysis of miRNA targets

**Fig. 5:**
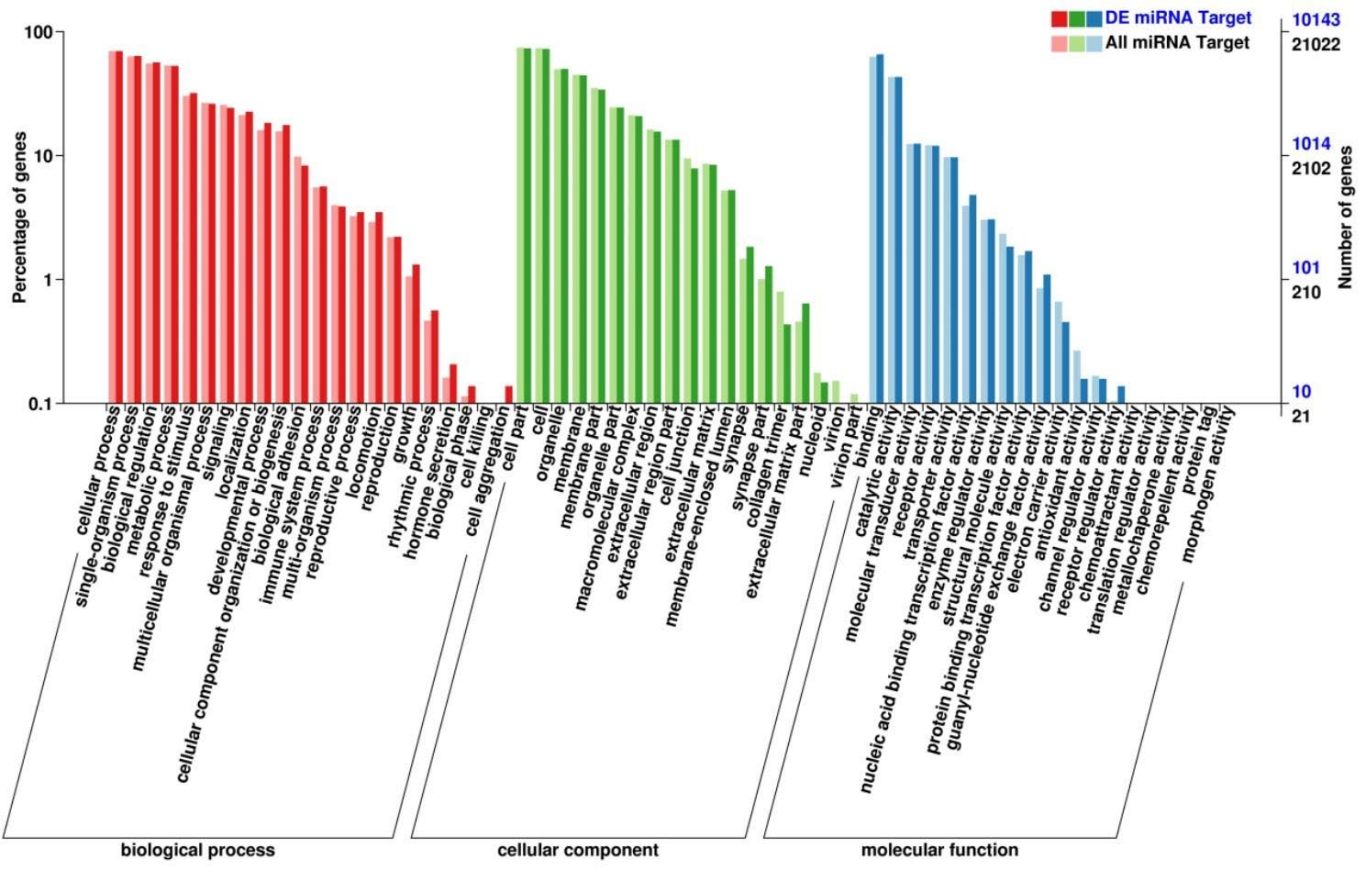
GO enrichment analysis for target genes of miRNA. red column: biological process; green column: cellular component; blue column: molecular function

GO analysis of target gene functions showed developmental process and reproductive process were enriched in biological process analysis, cell junction was enriched in cellular component analysis (Fig. 5; Supplementary Table 5).

**Fig. 6:**
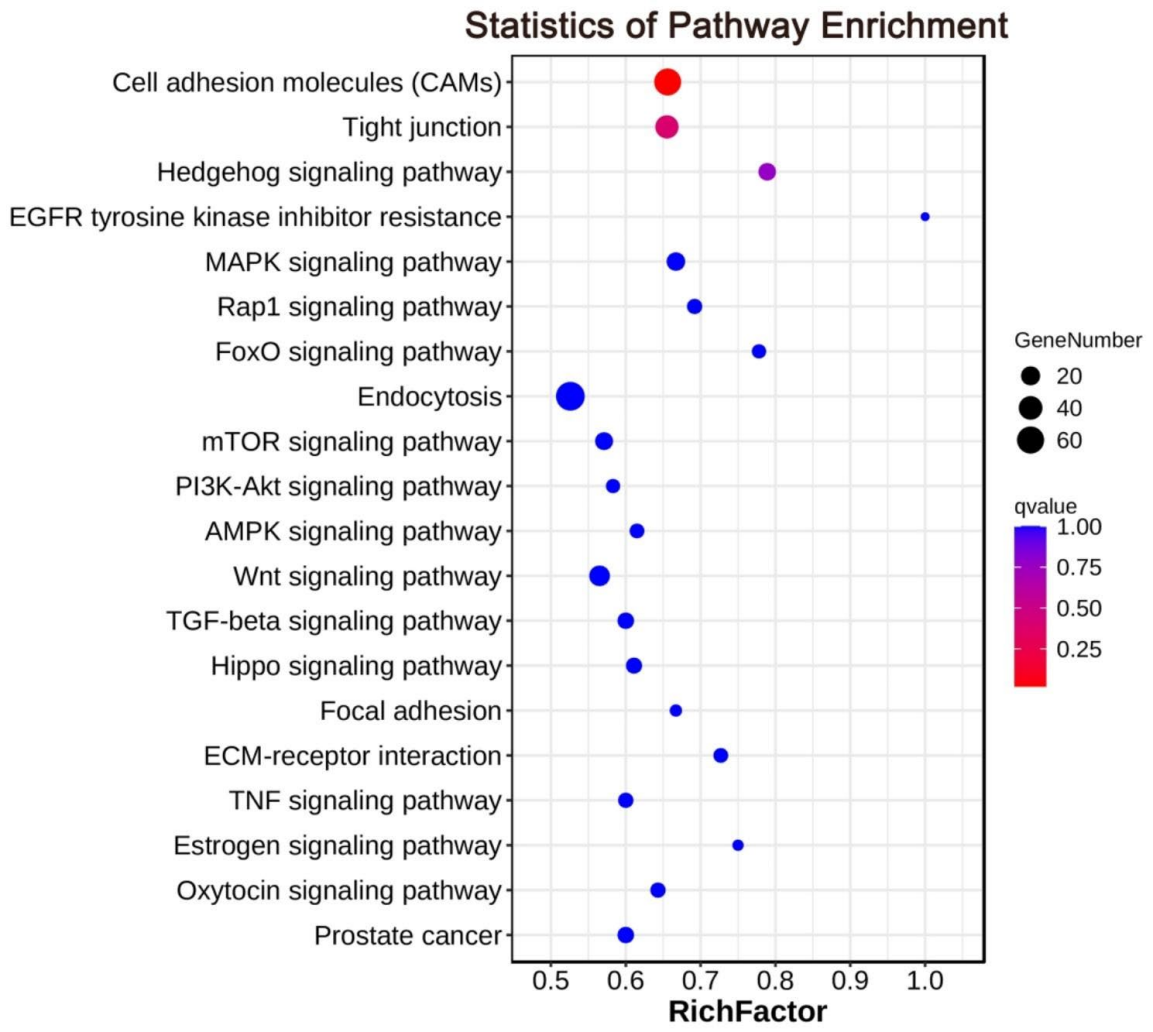
The top 20 KEGG pathways involved in reproduction of sheep. The size of point indicated the number of DE genes in pathway. The color of point indicated the range of qvalue

Results of KEGG pathway analysis revealed that DE miRNA targets were enriched in some pathways, including cell adhesion molecules (CAMs), tight junction, and other pathways involved in reproduction (Fig. 6; Supplementary Table 6). Especially, CAMs pathway was significantly enriched in KEGG analysis (qvalue < 0.05), which may participate in the regulation of sheep fecundity.

**Fig. 7:**
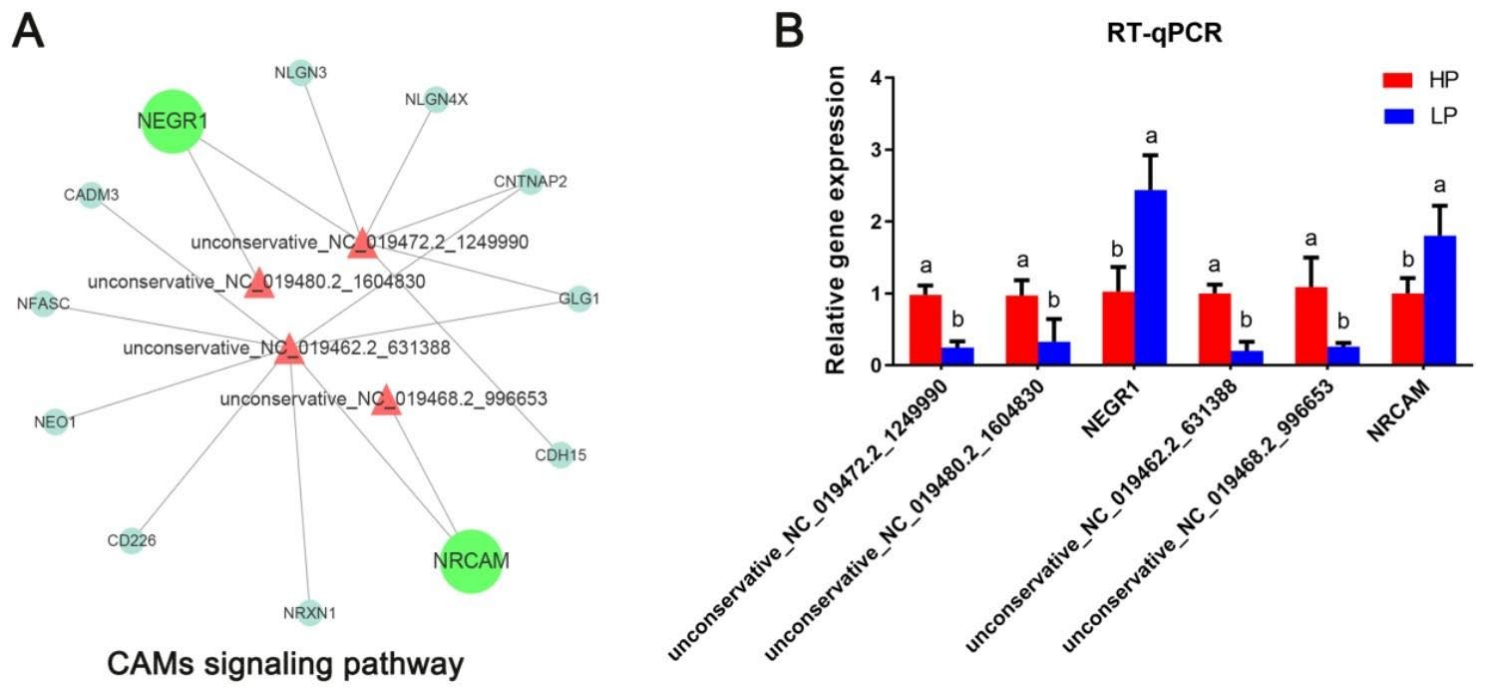
Analysis of miRNAs-mRNAs in CAMs signaling pathway. **(A)** The interaction network of miRNAs-mRNAs in CAMs signaling pathway [27–29]. red triangle: up-regulated miRNAs; green circle: down-regulated mRNAs; blue circle: no difference RNAs. **(B)** Analysis of miRNAs and mRNAs expression in CAMs by using RT-qPCR. The values are presented as means ± SDs. Means with different letters indicate significant differences (P < 0.05)

As shown in Fig. 7A, DE miRNAs (unconservative_NC_019472.2_1249990 and unconservative_NC_019480.2_1604830) were screened from CAMs signaling pathway, which targeted to *NEGR1*; DE miRNAs (unconservative_NC_019462.2_631388 and unconservative_NC_019468.2_996653) in CAMs signaling pathway targeted to *NRCAM*. Results of RT-qPCR showed the expression levels of miRNAs were negative connected with their predicted target genes (Fig. 7B).

## Discussions

The Hu sheep is famous for high prolificacy and year-around estrus, so it has attracted much attention in the mechanism research of sheep reproduction. As reported, uterus were important organs in prolific breeds of ewes that possess an intrinsically high ovulation rate as well as enhanced uterine capacity to maintain large litters [13]. Our previous study discovered the high prolificacy sheep had higher densities of uterine glands and endometrial microvessel, which is critical for enhanced endometrial uterine receptivity [30]. However, the molecular mechanisms underlying the effects of different uterine morphology on sheep reproductive performance remain unclear. At present, there are many reports focused on non-coding RNA in the regulation of endometrial functions [31, 32], and the expression pattern of miRNAs in ovine endometrium was identified demonstrated that miRNAs modulate ovine endometrium during the peri-implantation [33, 34]. Recent research identified miRNA transcriptome profile, and constructed miRNA-mRNA interaction in adrenal gland tissue of small tail Han sheep, discovered the miR-370-3p might play a major role in the reproduction regulation process [7]. Furthermore, the genome-wide analysis of ovarian miRNAs and mRNAs identified the roles of miRNAs in fecundity regulation between different sheep species [35]. To better understand the molecular mechanisms underlying the high prolificacy of Hu sheep, endometrial miRNAs sequencing was performed.

In present study, analysis of length distribution of endometrial miRNAs showed intensive enriching effects for 22 nt miRNAs in all samples, which was consistent with previous researches [36, 37]. Due to restriction site specificity, the first base of a mature miRNA sequence is highly biased. Therefore, we further analyzed the frequency distribution of bases at various positions of the endometrial miRNAs, which was found to be very similar to the typical miRNAs [38]. Previous study discovered differentially expressed miRNAs in ovaries were involved in the prolificacy of goat [12, 39, 40]. Research of miRNAs in sheep ovaries indicates miRNAs regulate the process of follicular development during the periovulatory stage, which provides new insights into the molecular mechanisms affecting sheep prolificacy [41]. Endometrial capacity is crucial for prolific breeds of ewes to maintain large litters [13]. However, the function of miRNAs in endometrium related to sheep uterine receptivity and prolificacy remains unknown. In order to clarify the role of endometrial miRNA in prolificacy of sheep, 58 differentially expressed miRNAs were screened in high prolificacy and low prolificacy sheep endometrium by RNA-seq in this study, especially the known miRNA oar-miRNA-136, oar-miRNA-154b-3p, oar-miRNA-410-5p, oar-miRNA-431, and oar-miRNA-665-3p. As reported, miRNA-136 significantly suppressed the expression of PPP2R2A in deciduas, these results further indicate that differentially expressed miRNAs may be involved in the pathogenesis of pre-eclampsia [42]. MiRNA-410-5p participates in the pathogenesis of abortion by regulating the biological function of trophoblast [43]. The dysregulation of miRNA-665 was implicated in the initiation and progression of endometrial cancer [44]. These researches signify that they may be extensively involved in sheep fertility regulation. Furthermore, some specific miRNAs were also discovered in sheep endometrium that may be potential markers affecting fertility. The accuracy of RNA-seq was validated by RT-qPCR. As previously reported, miRNAs can regulate the expression of their target genes via post-transcriptional regulation [45]. In order to analysis roles of DE miRNAs, RNA-seq were used to identify the potential miRNA-mRNA pairs, provided candidate targets for studying high-prolificacy traits in sheep. As study showed, estrogen receptor (ESR) gene was related to litter traits [46]. Among these miRNA-mRNA pairs, the core genes *ESR1* was identified the target of miRNAs unconservative_NC_019472.2_1229533 and unconservative_NC_019481.2_1637827. Furthermore, we also discovered unconservative_NC_019481.2_1637827 targeted to *TCF7*, which might play crucial roles in the molecular mechanism of the sheep prolific trait. As reported, the polymorphisms of TCF12 gene was related to litter size in pigs [47]. GO analysis of target gene discovered the developmental process, reproductive process, and cell junction were enriched. In the present study, the enriched KEGG pathways and GO pathways associated with reproduction clearly suggest that these miRNAs play a vital role in regulation of endometrial receptivity as well as the prolificacy of sheep. In present study, KEGG analysis of miRNA-targets revealed CAMs pathway as the candidate functional pathways was significantly enriched for high-prolificacy sheep. The major groups of CAMs involved in the embryo implantation are integrins, cadherins, selectins, immunoglobulins and mucins. These surface ligands mediate the adhesion between cells which are the maintenance of the structural integrity of tissue and receptivity of endometrium [48, 49]. These results indicated that CAMs may play a key role for high fecundity sheep by maintenance of endometrial receptivity. Furthermore, we discovered *NRCAM* and *NEGR1* in CAMs signaling pathway were significantly enriched in high prolificacy sheep as the targets of DE miRNAs. Previous research indicated NRCAM secreted by endometrial stromal cells, which enhanced the progestin sensitivity through epigenetic modulation [50]. Endometrial angiogenesis is closely related to the process of the cyclical development of the endometrium and embryo implantation [51]. NEGR1, as an inducible protein, is associated with vascular cells proliferation, differentiation, and death pathways [52]. Research also showed NEGR1 could be regulated by miR-150-5p in spinal cord ischemia–reperfusion injury model of rats [53]. However, the functions of miRNAs and their predicted targets analyses in sheep should be carefully evaluated by further experiments.

## Conclusion

In summary, this study provides the comprehensive analysis of endometrial miRNAs in Hu sheep with different fecundity, and discovered several target genes of miRNAs related to sheep prolificacy by the construction of miRNA-mRNA interaction network. In addition, CAMs signaling pathway was enriched in the endometrium of high prolificacy Hu sheep. Our study has crucial roles in understanding the regulatory mechanism of prolificacy in sheep.

### Electronic supplementary material

Below is the link to the electronic supplementary material.

Supplementary Material 1

Supplementary Material 2

Supplementary Material 3

Supplementary Material 4

Supplementary Material 5

Supplementary Material 6

Supplementary Material 7

## Data Availability

Data are available in the Sequence Reads Archive (SRA): [https://www.ncbi.nlm.nih.gov/sra/PRJNA964457].

## Abbreviations

CAMs: Cell adhesion molecules
ESR1: Estrogen receptorα
HP: High prolificacy
LP: Low prolificacy
miRNAs: MicroRNAs
NEGR1: Neuronal growth regulator 1
NRCAM: Neuron cell adhesion molecule
TCF7: Transcription factor 7
