# Supplementary material for: Identification and analysis of differentially expressed microRNAs in endometrium to explore the regulation of sheep fecundity
Source: BMC Genomics. 2023 Oct 9;24:600. doi: 10.1186/s12864-023-09681-y (PMC10563241; doi:10.1186/s12864-023-09681-y)
Supplement: Supplementary file 2 — Supplementary Material 2 [file 12864_2023_9681_MOESM2_ESM.docx]

Supplementary Table S1. Details of RT-qPCR primers

| Genes | Primer sequence (5′-3′) |
| --- | --- |
| unconservative_NC_019474.2_1351576 | F:CGGTGGTGCCAGCTCTGAG |
|  | R:AGTGCAGGGTCCGAGGTATT |
|  | RT:GTCGTATCCAGTGCAGGGTCCGAGGTATTCGCACTGGATACGA  CACTCTG |
| unconservative_NC_019477.2_1469651 | F:GCGCGTGGAATGTAAGGAAGT |
|  | R:AGTGCAGGGTCCGAGGTATT |
|  | RT:GTCGTATCCAGTGCAGGGTCCGAGGTATTCGCACTGGATACG  ACCCACAC |
| unconservative_NC_019462.2_655662 | F:GCGCAATTGTCTCCGTCTT |
|  | R:AGTGCAGGGTCCGAGGTATT |
|  | RT:GTCGTATCCAGTGCAGGGTCCGAGGTATTCGCACTGGATACG  ACTCTGAA |
| unconservative_NC_019458.2_21630 | F:GCGCGAACTGTGAGCTAGAG |
|  | R:AGTGCAGGGTCCGAGGTATT |
|  | RT:GTCGTATCCAGTGCAGGGTCCGAGGTATTCGCACTGGATACGA  CAATTCT |
| unconservative_NC_019484.2_1771114 | F:GCGCGCGCGTATATATATATATGT |
|  | R:AGTGCAGGGTCCGAGGTATT |
|  | RT:GTCGTATCCAGTGCAGGGTCCGAGGTATTCGCACTGGATACGA  CATACGT |
| unconservative_NC_019480.2_1592200 | F:CGCGTGGAGTGTGACAATG |
|  | R:AGTGCAGGGTCCGAGGTATT |
|  | RT:GTCGTATCCAGTGCAGGGTCCGAGGTATTCGCACTGGATACGA  CAAACAC |
| oar-miR-154b-3p | F:CGCGAATCATACATGGTTGAC |
|  | R:AGTGCAGGGTCCGAGGTATT |
|  | RT:GTCGTATCCAGTGCAGGGTCCGAGGTATTCGCACTGGATACGAC  AAAAAG |
| oar-miR-431 | F:CGTGTCTTGCAGGCCGTCA |
|  | R:AGTGCAGGGTCCGAGGTATT |
|  | RT:GTCGTATCCAGTGCAGGGTCCGAGGTATTCGCACTGGATACGAC  CCTGCA |
| unconservative_NC_019468.2_996653 | F:AACACGCGAGGGTTTGGGTTT |
|  | R:ATCCAGTGCAGGGTCCGAGG |
|  | RT:GTCGTATCCAGTGCAGGGTCCGAGGTATTCGCACTGGATACGACTCCCAC |
| unconservative_NC_019462.2_631388 | F:GCCGAGGCCCTTCCCTCCCG |
|  | R:ATCCAGTGCAGGGTCCGAGG |
|  | RT:GTCGTATCCAGTGCAGGGTCCGAGGTATTCGCACTGGATACGGGGAGGCG |
| unconservative_NC_019480.2_1604830 | F:TCGCGCATGACTCTGCAACC |
|  | R:ATCCAGTGCAGGGTCCGAGG |
|  | RT:GTCGTATCCAGTGCAGGGTCCGAGGTATTCGCACTGGATACGACCATGGG |
| unconservative_NC_019472.2_1249990 | F:AATCATGATTCTCCCCTTCCTCCC |
|  | R:ATCCAGTGCAGGGTCCGAGG |
|  | RT:GTCGTATCCAGTGCAGGGTCCGAGGTATTCGCACTGGATACGACCTGGGC |
| *NEGR1* | F:CGGACGCAGTGGATTGATAAGATG |
|  | R:CTGTACTTGGAGGGCTGAGAGG |
| *NRCAM* | F:ACAGCAGCAGGATCAGGAAGTC |
|  | R:TCTCAGCAGCGGCAGTAGTAAG |
| *ACTB* | F:TCAGCAAGCAGGAGTACGAC |
|  | R:ACGAGGCCAATCTCATCTCG |
| *U6* | F:CTCGCTTCGGCAGCACA |
|  | R:AACGCTTCACGAATTTGCGT |
