# Supplementary material for: Identification and analysis of differentially expressed microRNAs in endometrium to explore the regulation of sheep fecundity
Source: BMC Genomics. 2023 Oct 9;24:600. doi: 10.1186/s12864-023-09681-y (PMC10563241; doi:10.1186/s12864-023-09681-y)
Supplement: Supplementary file 7 — Supplementary Material 7 [file 12864_2023_9681_MOESM7_ESM.docx]

**Supplementary table legends**

**Supplementary Table 1. The quality control for miRNA sequencing.**

The raw data from RNA-sequencing needs to be purified for further analysis. Raw_reads, the Raw data from sequencing; Low_quality, reads (Quality score of base < 30) accounted for more than 20%; Containing'N'reads, reads containing at least 10% of the unknown base N; Length<18, reads smaller than 18 nucleotides; Length>30, reads larger than 30 nucleotides; Clean_reads, the number of reads of bases with Quality score > 30. Q30 (%), the proportion of reads with Quality score > 30.

**Supplementary Table 2. Details of RT-qPCR primers.**

The sequences of primers for RT-qPCR were listed in the table. F, forward primer; R, reverse primer; RT, reverse transcription primer of miRNA. ACTB and U6, the reference genes.

**Supplementary Table 3. The list of differential expression miRNAs.**

We screened out 58 differential expression miRNAs from high prolificacy sheep compared with low prolificacy sheep. The gene ID and expression levels of miRNAs from each sample were listed in table. |log2(FC)| > 1 and P-value < 0.05.

**Supplementary Table 4. The interaction network of miRNAs-mRNAs.**

The interaction network was analysed using miRanda and RNAhybrid based on the sequence information of miRNAs and mRNAs.

**Supplementary Table 5. GO analysis of predicted targets of miRNAs.**

GO enrichment analysis terms and numbers of genes were listed in the table. GO_classify1, primary classification of GO; GO_classify2, subclassificatio of GO; All miRNA Target, the number of genes in the specific GO term; DE miRNA Target, the number of differential expression target genes in the specific GO term; Total_gene, the number of target genes in all GO term list.

**Supplementary Table 6. KEGG analysis of predicted targets of miRNAs.**

KEGG pathways (TOP20) of target genes related to reproduction were listed in the table. Pathway, the name of KEGG term; ko, the ID of KEGG; enrichment_factor, The proportion of the number of differentially expressed miRNA target genes annotated to a pathway in the total number of genes annotated to that pathway. Q-value, the corrected P-value.
